# Training LSTM Networks With Resistive Cross-Point Devices

**DOI:** 10.3389/fnins.2018.00745

**Published:** 2018-10-24

**Authors:** Tayfun Gokmen, Malte J. Rasch, Wilfried Haensch

**Affiliations:** IBM Research AI, Yorktown Heights, NY, United States

**Keywords:** LSTM training, resistive processing unit (RPU), analog hardware, resistive device, resistive switching, DNN training, memristive device

## Abstract

In our previous work we have shown that resistive cross point devices, so called resistive processing unit (RPU) devices, can provide significant power and speed benefits when training deep fully connected networks as well as convolutional neural networks. In this work, we further extend the RPU concept for training recurrent neural networks (RNNs) namely LSTMs. We show that the mapping of recurrent layers is very similar to the mapping of fully connected layers and therefore the RPU concept can potentially provide large acceleration factors for RNNs as well. In addition, we study the effect of various device imperfections and system parameters on training performance. Symmetry of updates becomes even more crucial for RNNs; already a few percent asymmetry results in an increase in the test error compared to the ideal case trained with floating point numbers. Furthermore, the input signal resolution to the device arrays needs to be at least 7 bits for successful training. However, we show that a stochastic rounding scheme can reduce the input signal resolution back to 5 bits. Further, we find that RPU device variations and hardware noise are enough to mitigate overfitting, so that there is less need for using dropout. Here we attempt to study the validity of the RPU approach by simulating large scale networks. For instance, the models studied here are roughly 1500 times larger than the more often studied multilayer perceptron models trained on the MNIST dataset in terms of the total number of multiplication and summation operations performed per epoch.

## Introduction

Deep neural networks (DNNs) (LeCun et al., 2015) have made tremendous improvements in the past few years tackling challenging problems such as speech recognition (Hinton et al., 2012; Ravanelli et al., 2017), natural language processing (Collobert et al., 2012; Jozefowicz et al., 2016), image classification (Krizhevsky et al., 2012; Chen et al., 2017), and machine translation (Wu, 2016). These accomplishments became possible thanks to advances in computing resources, availability of large amounts of data, and clever choices of neural network architectures. For instance, spatial correlations in the data are exploited by convolutional neural networks (CNNs) (LeCun et al., 1988; Krizhevsky et al., 2012; He et al., 2015) whereas temporal correlations can be handled by recurrent networks (Lipton et al., 2015). One of the most common recurrent architectures is long short-term memory (LSTM) (Hochreiter and Schmidhuber, 1997; Karpathy et al., 2016). LSTMs in combination with CNNs provide end-to-end trainable building blocks for composing complex neural network architectures, that are used for challenging tasks such as image captioning (Karpathy and Fei-Fei, 2015). Training these large complex DNNs are extremely computational intensive tasks and today most of these workloads run on general purpose digital hardware such as CPUs and GPUs in a massively parallel fashion (Dean et al., 2012; Coates et al., 2013; Chilimbi et al., 2014; Gupta et al., 2017). There are many attempts to accelerate training of large scale DNNs by designing and using specialized digital hardware (Emer et al., 2016), such as Google’s TPU (Jouppi et al., 2017) or Intel’s KNL (Sodani, 2015), relying on optimized multiplication and summation operations. In addition to the digital approaches, resistive cross-point device arrays are also promising candidates that perform the multiplication and summation operations in the analog domain which can provide massive acceleration and power benefits (Gokmen and Vlasov, 2016).

The concept of using resistive cross-point device arrays (Burr et al., 2015, 2017; Chen et al., 2015; Prezioso et al., 2015; Agrawal et al., 2016b; Gokmen and Vlasov, 2016; Fuller et al., 2017) as DNN accelerators has been tested, to some extent, by performing simulations for the specific cases of fully connected (Gokmen and Vlasov, 2016) and convolutional neural networks (CNNs) (Gokmen et al., 2017). The effect of various device properties and system parameters on training performance has been evaluated to find the required device and system level specifications for a successful implementation of an accelerator chip that efficiently trains DNNs (Agrawal et al., 2016a; Gokmen and Vlasov, 2016). As shown empirically by simulations (Agrawal et al., 2016a; Gokmen and Vlasov, 2016; Gokmen et al., 2017), a key requirement is that these analog resistive devices must change conductance symmetrically when subjected to positive or negative pulse stimuli. Indeed, this requirement differs significantly from those needed for memory elements and therefore require either an additional circuit overhead (Ambrogio et al., 2018) or systematic search for new physical mechanisms, materials and device designs to realize such an ideal resistive element for DNN training. In addition to these critical device properties, the peripheral circuits and the whole system needs to be designed carefully within the specifications for successful DNN training. For instance, noise and signal saturation inherent to the analog nature of the computations performed on the arrays limit the training accuracy of CNNs on MNIST dataset (Gokmen et al., 2017). As a solution number normalization and signal bound detection techniques are proposed (Gokmen et al., 2017) and these techniques may be incorporated into the system design.

It is clear that resistive cross-point devices, so called resistive processing unit (RPU) (Gokmen and Vlasov, 2016) device arrays that can simultaneously store and process data locally and in parallel, are promising candidates for intensive DNN tasks computations; however, any future hardware that is targeting DNN applications needs to be able to offer solutions for handling a range of network architectures including fully connected, convolutional as well as recurrent layers. Here, we extend the application space of RPUs to recurrent neural networks (RNNs). We show how to map the complex recurrent LSTM blocks to RPU arrays and test the effect of various device level imperfections and peripheral circuit constraints, such as input signal resolution, to the training accuracy of LSTM networks on a character based language model. We also study the effect of dropout during training LSTMs in the presence of device imperfections and system level constraints. Although, dropout is critical when training large LSTMs with floating point (FP) numbers to mitigate overfitting, it turns out that for RPU simulations training is not significantly affected by dropout. This suggests that some of the device imperfections and noise in the hardware may act as a regularization term during training. However, we also show that among all device variations the requirement for having a very accurate match in the average minimal up and down update step sizes throughout the training process for each cross-point device (symmetric updates) become increasingly more important and any mismatch needs to be minimized for successful training. Our results further emphasize the importance of device symmetry when realizing a resistive element suitable for DNN training.

## Materials and Methods

### LSTM Block

**FIGURE 1:**
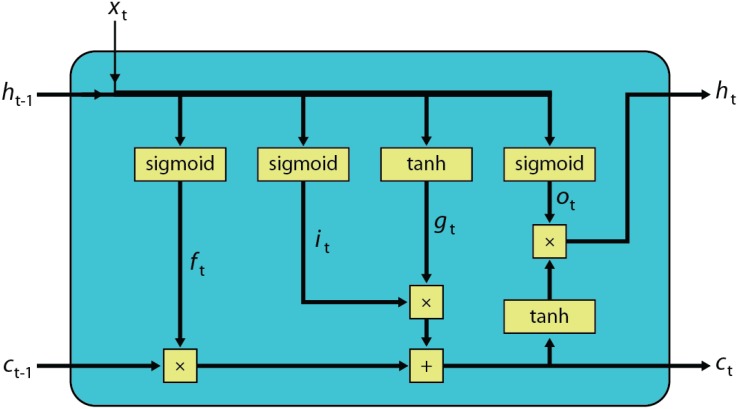
Computational graph of a LSTM block.

The dynamics of an LSTM block (Hochreiter and Schmidhuber, 1997) is described using deterministic transitions from previous state to current state are given by the equations below (see also the corresponding computational graph in Figure 1):

(1) $$f_{t}=\text{sigmoid}(W_{f}x_{t}+U_{f}h_{t-1}+b_{f})$$

(2) $$i_{t}=\text{sigmoid}(W_{i}x_{t}+U_{i}h_{t-1}+b_{i})$$

(3) $$o_{t}=\text{sigmoid}(W_{o}x_{t}+U_{o}h_{t-1}+b_{o})$$

(4) $$g_{t}=\tanh(W_{g}x_{t}+U_{g}h_{t-1}+b_{g})$$

(5) $$c_{t}=f_{t}\times c_{t-1}+i_{t}\times g_{t}$$

(6) $$h_{t}=o_{t}\times \tanh(c_{t})$$

where *x_t_* is the input vector of length *n* for the current time step *t, h*_*t*−1_ and *h_t_* are the hidden state vectors, *c*_*t*−1_ and *c_t_* are the memory state vectors of length *m* from the previous and current time steps, respectively. The trainable parameters for the LSTM block are stored in *W_f_, W_i_, W_o_, W_c_* matrixes of sizes *m* × *n, U_f_, U_i_, U_o_, U_c_* matrixes of sizes *m* × *m* and bias terms *b_f_, b_i_, b_o_, b_c_* of sizes *m* × 1. *f_t_, i_t_, o_t_* and *g_t_* respectively, correspond to the forget gate, input gate, output gate, and new candidate memory state, all of which are vectors of length *m*. In these equations sigmoid and tanh functions are applied element-wise and × is an element-wise multiplication (Hadamard product).

Just like regular feed forward networks, LSTM networks are trained using the backpropagation algorithm (Rumelhart et al., 1986). However, the concept of time in the case of an LSTM is simply expressed by a well-defined ordered series of calculations linking one time step to the next one and therefore error signals are backpropagated through time (*BPTT*) (Hochreiter and Schmidhuber, 1997). The number of unrolling steps through time used for backpropagation, *n_BPTT_*, is a hyperparameter of the LSTM training. During training, all activations calculated during the forward pass for each time step need to be stored for the backward pass (for the calculation of the derivatives). Once the backward pass is completed for all *n_BPTT_* time steps, the total weight change, which is the sum of the gradients from each time step, can be calculated and applied to update the weights. Similar to the weight sharing concept for convolutional layers at different spatial locations, for an LSTM block, the weights are shared between different time steps and the amount of sharing is controlled by the choice of *n_BPTT_* during training.

### Mapping of an LSTM Block to Resistive Device Arrays

**FIGURE 2:**
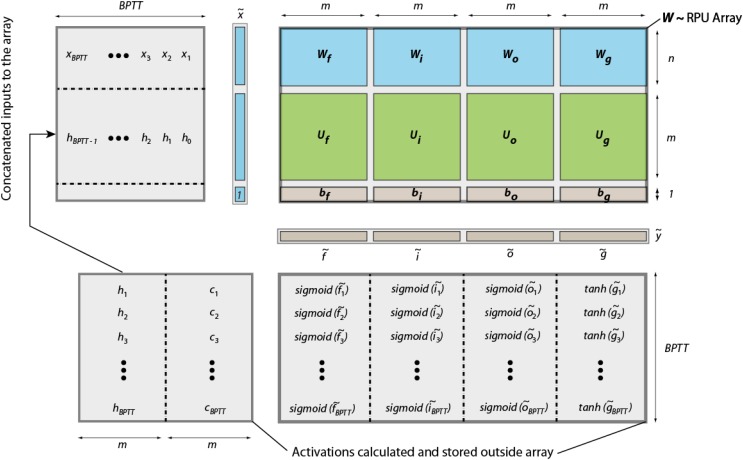
Schematics of an LSTM block mapped to an RPU array. The input vectors to the RPU array and the output vectors from the array are shown for the forward pass only. All activations are calculated and stored outside the array by digital NLF circuits. We note that each step of the computation happens sequentially in time, but results are also stored spatially at different locations in the memory.

Figure 2 illustrates all the calculations that need to be performed for an LSTM block during a forward pass and their mapping onto an RPU array. All trainable parameters of an LSTM block can be organized into a single matrix *W* of size 4*m* × (*m* + *n* + 1) which is then mapped onto a single RPU array of the same size. We denote the temporary input vector to the RPU array, that is used for each time step, as 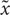, which is the concatenated vector of the input vector from current time step *x_t_*, the hidden state vector from the previous time step *h*_*t*−1_ and a single bias value of unity. Performing a single vector-matrix multiplication 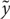 = W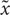 yields a vector 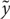 of length 4*m* where different portions can be used to calculate activations given by Eqs (1–4) for a single time step. We note that the single 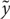 = W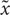 operation completes all the linear transformations that are needed and has the computational complexity of *O*(4*m* × (*m* + *n* + 1)). It can be performed with *O*(1) time complexity once mapped to RPU arrays thanks to the array parallelism. All other computations shown above are point wise operations and therefore have the computational complexity of *O*(*m*) + *O*(*n*). We assume this part of the computations are performed outside of the RPU array by a digital block, the so-called non-linear function unit (NLF) (Gokmen and Vlasov, 2016). We note that all steps shown by Eqs (1)-(6) are repeated *n_BPTT_* times before error backpropagation starts; and similar computation steps are performed during the error backpropagation starting from the last time step. For instance, in the backward pass the computations that are performed on the array can be written as 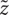 = *W^T^*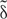, where 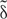 is the temporary error signal generated at each time step and *W^T^* is the transpose of the original weight matrix used during the forward pass. The 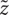 vector is further processed by the NLF units so that the error signal for the previous time step can be generated. Once the backward steps are repeated *n_BPTT_* times, the weight update can be written as a series of updates *W* ← *W* + η(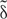

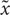
^*T*^). These are again performed *n_BPTT_* times for the *n_BPTT_* 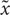 and 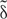 vectors corresponding to each iteration step during the forward and backward pass. Therefore, a LSTM block can be viewed as a fully connected layer but with parameter sharing that happens between time steps by reusing the same weight matrix for each step of the calculation. We emphasize that all other non-linear operations of the LSTM block are performed by the NLF units outside of the array; and these NLF units require an access to a local or an external storage (memory) in order to save the intermediate results computed during the forward (and backward) pass that are also needed during the error calculation at backward pass (and update cycle).

### System Level Simulation Details

In a real system, all computations that are needed to train a neural network have to be handled by some sort of underlying hardware. Here, we assume a system that is composed of analog arrays and digital NLF blocks. Each operation necessary for the training of a multilayer LSTM network is mapped to either the analog or digital parts of the system. If an operation is handled by the analog unit, we simulate the corresponding operation using the limitations of the underlying analog hardware. For instance, all of the vector-matrix operations involving the weights during forward, backward, and update cycles are mapped onto analog arrays. Therefore, these computations are performed using various hardware defined constraints as described in more detail in Section “Results.” All other operations such as sigmoid, tanh, Hadamard product, point-wise summation and softmax are mapped to the digital NLF blocks. We assume that these digital units perform the operations using 32-bit FP numbers and do not introduce any additional non-idealities, such as noise and quantization errors, beyond the ones introduced by analog units. We also assume that the results computed by the digital NLF blocks during forward (or backward) cycle are stored in a standard memory in 32-bit FP format and that these results are accessed later during backward (or update) cycle without any loss of information. In order to estimate the training accuracy achievable by such a system, we developed a simulation tool (written in C++) that trains the neural network based on these described constraints, and in particular, simulates the hardware defined limitations of the analog units at every stage of the neural network training.

## Results

In order to test the validity our approach to map LSTMs to RPUs, we train LSTM networks similar to those described in Karpathy et al. (2016), composed of 1 or 2 stacked LSTM blocks, with different numbers of hidden vector sizes of 64, 128, 256, or 512 on two datasets: Leo Tolstoy’s War and Peace (WP) novel and Linux Kernel (LK) consisting of 3,258,246 and 6,448,461 characters, respectively. We split the data into training and test sets as 2,933,246 and 325,000 characters for WP and 6,111,421 and 337,040 characters for LK where each dataset, respectively, have a total vocabulary of 87 and 101 characters. Throughout the paper we use the following naming convention consisting of the network block, stacking, hidden vector length and the dataset. For instance LSTM2-512-WP is a 2 stacked LSTM network with a hidden vector size of 512 trained on the WP dataset. Following the mapping described above for LSTM2-512-WP, we use three different arrays with sizes 2048 × (512+87+1) and 2048 × (512 + 512 + 1) for the two LSTM blocks and a third array of size 87 × (512 + 1) for the last fully connect layer before the softmax activation (softmax layer as a whole). We note that the total number of trainable parameters for the largest networks trained here is about 3.4*M* and the total number of multiplication and summation operations that needs to be performed during a single training epoch is about 10^14^. These large number of operations makes these simulations about 1500x more challenging than training a fully connected network on the MNIST dataset (Gokmen and Vlasov, 2016).

### Optimization Approach

It is critical to perform training simulations that can be supported by real RPU array hardware. Although operations performed on the RPU array during the update cycle are all computed in parallel, RPU arrays only support operations of the form

(7) $$W\leftarrow W+\eta (\tilde{\delta }{\tilde{x}}^{T})$$

which is an outer product of two vectors and a weight update combined into a single operation. This form is consistent with the simple SGD rank-1 update but any variant of a SGD such as RMSProp, Adagard, momentum, etc., all require the calculation of the gradient values first and then updating the weight value using some history dependent parameter per weight that is a function of previous weight values and/or gradients. In its most general form these operations can be written as a two-step process.

(8) $$\Delta W=\tilde{\delta }{\tilde{x}}^{T}$$

(9) $$W\leftarrow W+\eta (W_{\mathrm{pre}},\;\Delta W_{\mathrm{pre}})\Delta W$$

where the first operation is the gradient calculation and the second is the weight update. On a digital hardware, the computation overhead of these additional calculations may be insignificant and can easily be implemented by storing and updating one additional parameter per weight and do not increase the computational complexity. However, for RPU arrays such an extra operation will break the array parallelism as the update cannot be performed at constant time anymore. While the calculations of the gradients given by Eq. (8) can still be performed on a separate array with *O*(1) time complexity, Eq. (9) can only be implemented column-wise serially with *O*(*m*) time complexity and therefore violates the array parallelism.

**FIGURE 3:**
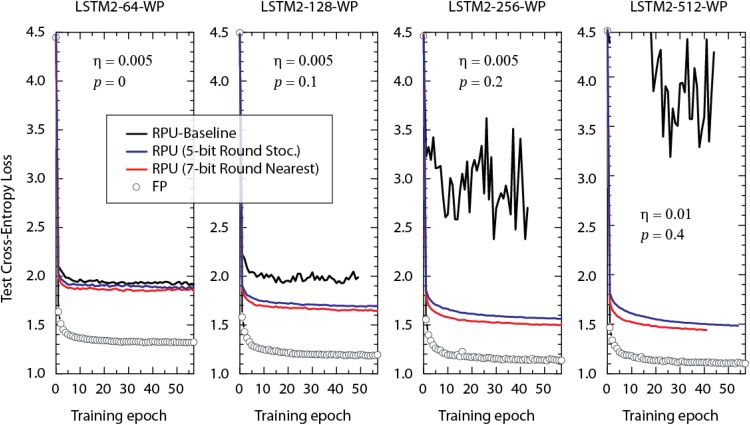
Test cross-entropy loss of two stacked LSTM networks (with different hidden vector sizes) trained on the WP dataset. Open white circles correspond to the model where the training is performed using floating point (FP) numbers. Lines with different colors correspond to RPU-baseline models using different input signal resolutions and rounding schemes as given by the legend. Same dropout probability, *p*, and the learning rate, η, are used for the FP and RPU models for each network size. For the sake of comparison we did not optimize these parameters with respect to the RPU model.

In order not to violate parallel array operations, in our simulations training is performed using only simple SGD. Additionally, mini-batch size of unity, fixed learning rate, and time unrolling steps *n_BPTT_* of 100 is used. Since these settings are slightly different from what is used in Karpathy et al. (2016) (such as RMSProp with mini-batch size of 100), we first validated our training by performing simulations using standard cross-entropy loss function and high precision FP numbers/operations and tried various learning rates, η, with different amount of dropout rates, *p*, for each model individually to reach the performance levels reported by Karpathy et al. (2016). We note that the dropout term is only introduced for non-recurrent connections following the guidelines from (Zaremba et al., 2014) and is consistent with (Karpathy et al., 2016). Figure 3 shows the best baseline-FP results for various LSTM2-WP models with different hidden vector sizes at the corresponding learning rate and dropout rates. For each model the test cross-entropy loss is on par or slightly better than the value reported by Karpathy et al. (2016) and therefore validates our simple SGD training approach.

### RPU Baseline Model

The various RPU device imperfections and their effect on the training accuracy were tested previously for a fully connected (Gokmen and Vlasov, 2016) and a convolutional (Gokmen et al., 2017) neural network on the MNIST dataset. Although the same device specifications were sufficient to train both networks successfully, input/output signal normalization/renormalizations were needed for successful training of CNNs. Here in our simulations we start with a baseline model that has identical device parameters and signal normalization techniques as described for CNNs (Gokmen et al., 2017).

The RPU-baseline model uses a stochastic update scheme (Gokmen and Vlasov, 2016), where the length of the stochastic stream is *BL* = 10. The gain factors *C*_x_ and *C*_δ_ used for determining the pulse probability during the stochastic translation for the columns and the rows are scaled properly (*C*_x_ = *C*_δ_ = $\sqrt{\eta /\mathrm{BL}\Delta w_{\min})}$) to give the desired learning rate, η, used for training the model; and Δ*w*_min_ is the average incremental change in the weight value due to a single coincidence event. Although the average value for Δ*w*_min_ is set to 0.001, in order to capture device imperfections, Δ*w*_min_ is assumed to have cycle-to-cycle and device-to-device variations of 30%. Possible asymmetry in the weight updates are taken into account by using separate $\Delta w_{\min}^{+}$ for the positive updates and $\Delta w_{\min}^{-}$ for the negative updates for each RPU device. The average value of the ratio $\Delta w_{\min}^{+}/\Delta w_{\min}^{-}$ among all devices is assumed to be unity but with a 2% device-to-device variation. The bounds on the weights values, |*w_ij_*|, are set to be 0.6 on average with a 30% device-to-device variation. For any real hardware implementations of RPU arrays the results of the vector matrix multiplications will be noisy and this noise is considered by introducing an additive Gaussian noise, with zero mean and standard deviation of σ = 0.06. Furthermore, the results of the vector-matrix multiplications are bounded to a value of |α| = 12 to account for signal saturation. The input signals are assumed to be between [−1, 1] with a 5-bit input resolution, whereas the outputs are quantized assuming a 9-bit ADC. Although the input signals going into the array and the output signals coming from the arrays are bounded, we use noise management and bound management techniques described in Gokmen et al. (2017). In particular, the inputs/outputs are normalized/renormalized using to the absolute maximum value of the elements of vectors 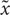 or 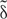 during the forward and backward passes, respectively. These normalizations are crucial not only because of small backpropagating error signals as discussed in Gokmen et al. (2017) but also during forward propagation, because values in 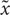 can go beyond unity due to the dropout term used: note that during training time when dropping a random fraction *p* of activations, the remaining are scaled with 1/1 − *p* and therefore the input signals might become larger than 1.

The test error of this RPU-baseline on various LSTM2-WP models with different hidden vector sizes are shown in Figure 3 as black curves. Each model uses the same learning and dropout rates as for the corresponding floating point reference model (FP-model). In contrast to the behavior observed for the FP-models, test errors of the RPU-baseline models increase and become noisier when the size of the network is enlarged. This is a very disappointing scaling result and if not addressed properly, may limit the application space of analog device arrays to only a very small network sizes.

In order to identify the main cause of this problem, we performed training at various training conditions. For the models that are trained with a larger input signal resolution of 7-bits (but otherwise identical device and system properties), as shown by red curves in Figure 3, the test error follows a trend much more similar to the FP-model. Although, there remain some offsets between the FP-models and the RPU-models trained with 7-bit input resolution, offsets tend to get smaller and RPU-models improve in performance as the number hidden vector size (or parameters) increases. These results show that the undesired behavior observed for the black curves (RPU baseline) are solely due to the limited input signal resolution of 5-bits.

### Stochastic Rounding of Input Signals

It is clear that the limited input signal resolution needs to be addressed for successful application of the RPU approach on large networks, however, increasing the input signal resolution comes with a cost of increased peripheral circuit complexity or computation time. For instance, for time encoded signals, increasing the input resolution from 5 to 7-bits increases signal duration by a factor 4 for the largest input, and therefore increases the computation (integration) time during forward and backward passes. Alternatively, for a fixed integration time, 7-bit inputs require four times faster clock rates during signal generation and therefore it may not be possible given the limitations due to signal filtering and clock rates. Using voltage height controlled inputs also comes with a cost, as more voltage levels need to be generated by the peripheral circuits which again increases the circuit complexity.

Here, we propose to use a stochastic rounding scheme (instead of rounding to nearest neighbor) as a cost effective solution for recovering the test accuracies while still keeping signal resolution at 5-bits. It is already shown that stochastic rounding helps during DNN training when used at different stages of approximate computing with reduced precision in the digital space (Gupta et al., 2015). However, to prove the effectiveness of stochastic rounding for training RPU arrays, we performed simulations using the same RPU-baseline model with 5-bit input resolution but instead with the stochastic rounding scheme. As shown by the blue curves in Figure 3, stochastic rounding at 5-bit input resolution almost completely recovers the results of a round-to-nearest-neighbor scheme at 7-bits and therefore it can be a viable approach for real hardware implementations. The overhead of using stochastic rounding instead of rounding to nearest neighbor is very small (Gupta et al., 2015) and it can be realized by specifically designing additional hardware residing in the digital blocks that moves data between RPU arrays and NLF units. Although our simulations do not guarantee that the 5-bit input resolution is sufficient for even larger networks, our results nevertheless suggest that using stochastic rounding saves a couple of bits during input signaling and hence improves the overall performance of the RPU arrays.

### Effect of Dropout

**FIGURE 4:**
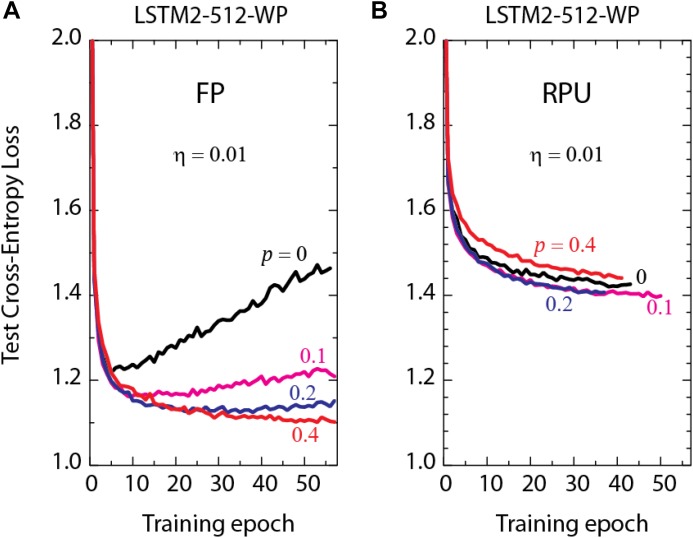
Test cross-entropy loss of two stacked LSTM networks with a hidden vector size of 512 trained on WP dataset. Lines with different colors correspond to **(A)** the model trained using FP numbers **(B)** the RPU-baseline models using 7-bit input signal resolution, at different dropout probabilities, p.

It is known that successful applications of large neural networks require good regularization and dropout (Srivastava et al., 2014) is one of the most powerful regularization methods. Indeed, we also use dropout in our training and larger dropout rates are needed for the best performance as the network size gets larger as shown by the FP-models in Figure 3. To highlight the importance of dropout, we show the test results of LSTM2-512-WP, the largest network of interest, trained at different dropout probabilities in Figure 4A. It is clear that small dropout rates (*p* < 0.4) cause the networks to overfit since the test errors start to increase after a certain amount training. Only the cases with a 40% dropout rate or higher show a consistent downward trend in the test errors and hence do not suffer from the overfitting problem. However, increasing the dropout rate arbitrarily beyond 40% is not beneficial either (data not shown) and the best generalization results are observed at about 40% dropout rates for LSTM2-512-WP when trained with FP numbers.

In order to test the effect of dropout for a realistic hardware implementations of RPU arrays, we performed training using the RPU-baseline model at 7-bit input resolution and varied the dropout rates, as shown in Figure 4B. In contrast to the results obtained by the FP-models, even when dropout is not used at all, we did not observe overfitting to be a problem. In addition, the best performance is obtained for dropout rates at around 10–20%, which is smaller than the optimal value used for FP-models at 40%. These results suggest that for a realistic implementation of RPU arrays the training may not require such a strong regularization term or the same amount may be non-optimal as there exists many sources of noise and stochasticity coming from the hardware. However, it is also important to realize that the effect of the dropout is much smaller for all RPU models; and even with the optimal dropout rates we consistently observe an offset between the RPU-models and FP-models for all LSTM sizes (data for smaller networks are not shown).

### Effect of Device Variations, Asymmetry, and Number of States

**FIGURE 5:**
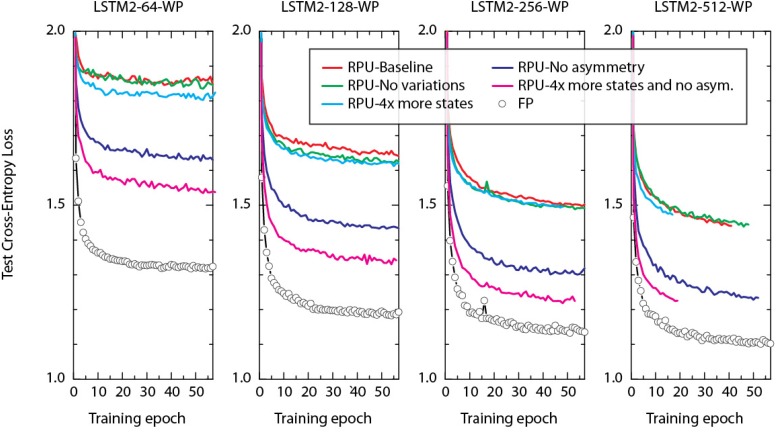
Test cross-entropy loss of two stacked LSTM networks (at different hidden vector sizes) trained on WP dataset. Open white circles correspond to the model where training is performed using FP numbers. Simulation of RPU-baseline models are shown by red curves. Lines with different colors correspond to RPU models but for each model a set of device imperfections are selectively eliminated compared to the RPU-baseline model. Green curves: device-to-device and cycle-to-cycle variations in the parameters Δ*w*_min_ and |*w*_ij_| are completely eliminated. Cyan curves: the total number of states is increased by 4x. Blue curves: device-to-device variation in the asymmetry parameter is eliminated. Magenta curves: as for blue curves, but additionally the number of states are increased by 4x. All RPU models are trained with 7-bit input signal resolution using round-to-nearest-neighbor scheme. Since the simulation run times were limited to 7 days, some curves stop early before reaching 50+ epochs.

To understand the main cause of the offset observed between the FP and RPU models we performed training using a range of RPU models. In each we selectively eliminated device imperfections to evaluate their influence on training performance. The summary of these training results on LSTM2-WP with different hidden vectors sizes are shown in Figure 5. The green curves in Figure 5 correspond to the models where device-to-device and cycle-to-cycle variations in the parameters Δ*w*_min_ and |*w_ij_*| are completely eliminated from their original values at 30%. Interestingly, eliminating of all this variability from the model does not improve the network performance compared to the RPU-baseline models as shown by red curves. Similarly, the cyan curves corresponding to RPU models with a larger number of states (4x more compared to baseline) also show test errors that are almost indistinguishable from the RPU-baseline model. Only the blue curves corresponding to RPU models without any device-to-device variation in the asymmetry parameter ($\Delta w_{\min}^{+}/\Delta w_{\min}^{-}$) show an improvement and test errors get closer to the value achieved by the FP models (shown by open circles). These results suggest that the most important factor limiting the performance of the RPU models is the asymmetry parameter and even a slight asymmetry term with only a 2% device-to-device variation is sufficient to be harmful. Only after the elimination of the asymmetry term, increasing the number of states further enhances the network performance as shown by magenta curves. We note that all RPU models shown in Figure 5 are simulated using a 7-bit input resolution in order not to introduce additional artifacts due to limited input resolution; and each model used the same dropout and learning rate that the FP-model is trained with.

## Discussion and Conclusion

In this study, we explored the applicability of the RPU device concept to training LSTMs. We found that training LSTM blocks is very similar to the training of fully connected layers, because a single vector operation can be used to perform all linear transformations needed for a single time step in the LSTM training.

The operations performed on the arrays are identical for different network architectures including fully connected, convolutional or recurrent networks. However, it is not obvious that the same device constraints derived from a small fully connected network can be generalized to give competitive training accuracies for larger and more complex networks on larger datasets. Studying LSTM networks here is an attempt to generalize the insights regarding the effect of different hardware noise and device variations on the training performance gained previously by investigating relatively small learning tasks to a much more challenging learning task. The array sizes used for the LSTM2-512-WP model are (2048 × 600), (2048 × 1029) and (87 × 513) and these arrays are much larger than the ones used for the fully connected network studied in Gokmen and Vlasov (2016) with sizes of (256 × 785), (128 × 257), and (10 × 129). In addition, the training sequence consists of about 3M characters for the WP dataset compared to 60K training images in the case of the MNIST dataset. The combination of larger array sizes and more training examples makes the training of these LSTM networks about 1000x (1500x for LK dataset) more challenging than training the MNIST dataset on the aforementioned fully connected network. **Interestingly, the 2% variation in the asymmetry term that was sufficient to train the fully connected network at the level of FP model accuracy is shown to be not sufficient for these LSTM networks**. This result suggests that the asymmetry parameter becomes increasingly more critical for larger scale networks and it may require special attention during hardware design and development.

The performance benefits of the RPU approach for LSTM networks can be calculated using the design considerations described in Gokmen and Vlasov (2016). For an LSTM block, computation steps depend on the computations in previous time steps and, if stacked LSTM networks are used, additionally on computations in the previous LSTM blocks. Therefore, a pipelined microarchitecture design is required to utilize multiple RPU arrays that can compute concurrently on multiple overlapping time steps or data. Assuming a fully pipelined architecture and the LSTM2-512-WP model, there would be a total of 3.4M RPU devices active at any given time residing on three different arrays. Using a measurement (cycle) time of roughly *t*_meas_ = 80*ns* (Gokmen and Vlasov, 2016) for each forward, backward and update cycles, we can estimate the total RPU accelerator chip performance using the below simple formula

(10) $$\text{Throughput}=\frac{2\times \mathrm{Total}\_\mathrm{RPU}\_\mathrm{Count}}{t_{\text{meas}}}\mathrm{Ops}/s$$

where the factor of two comes from the multiplication and the summation operations performed on each RPU device. This yields a throughput of 85*TeraOps/s* for the LSTM2-512-WP model. This is already significantly higher than the peak single precision throughput of an NVIDIA Tesla P100 at about 10*TeraOps/s*. Thus the performance benefits of the RPU approach becomes already apparent for the sizes of the LSTMs investigated here, and once it is applied to much larger problems with a total number of RPU devices reaching billions, throughput of an RPU accelerator chip exceeds the throughput of todays’ advanced GPUs and accelerators by more than 1000x.

The concepts described in this study can also be applied to more complex neural networks including a mixture of recurrent, convolutional and fully connected layers by simply reprogramming digital blocks that compute the non-linear functions and control the signal flow. Note that we assume that all other non-linear operations are performed outside of the RPU arrays by using programmable digital circuits and that the results are sent back to the same RPU array for the next step of the calculations. For LSTMs, this realizes the weight sharing that happens between different time steps, but this makes the architecture also flexible to implement other heterogeneous network architectures. Since these programmable digital blocks control the signal flow and can perform any type of computation, it becomes also very easy to implement other kinds of recurrent networks on the same hardware. For instance gated recurrent units (GRUs) (Cho et al., 2014), dilated RNNs (Chang et al., 2017) or other more complex RNN architectures (Chung et al., 2015) can be mapped to RPU arrays in a similar fashion by simply changing the computations performed on the digital circuits.

In order for RPUs to be a competitive technology, however, the symmetry requirement of the weight update needs to be addressed. Accomplishing such symmetrically switching analog devices as needed is a difficult task. Besides material engineering, circuit assisted solutions combined with algorithmic modifications might, conceivably, relax the material requirements. One example of an almost perfectly symmetric RPU is demonstrated by designing analog CMOS (Kim et al., 2017; Li et al., 2018) (so called CMOS-RPU) that performs the updates using a current source and sink circuitry and stores the weight as charge on a capacitor. In this design, it is shown that symmetry is achieved by properly balancing the current source and sink that incrementally change the stored charge on the capacitor. Device leakage, device mismatch and charge retention on the capacitor are critical components for the scalability to larger networks. Functionality of this RPU concept comes at the cost of significant circuit overhead. In contrast to the CMOS-RPU approach, there are device options available that may be used to realize the RPU concept. One noteworthy device concept is the so called LISTA device (Fuller et al., 2017) that shows significantly more symmetric behavior if a current pulsing scheme is used. However, this current pulsing scheme would also require a current source and sink circuitry similar to the ones used in the CMOS-RPU design. A simple constant voltage pulsing scheme is difficult to realize for the demonstrated LISTA devices in an array configuration due to the built-in voltage which depends on the individual weight state of each node. By properly selecting the materials used in the device stack this built-in voltage problem can be mitigated and it is an interesting research direction for realizing a symmetric RPU concept. Finally, we note that PCM devices (Burr et al., 2015, 2017) are promising candidates to realize the RPU concept. PCM elements change their conductance gradually at one polarity (SET) and very abruptly at the opposite polarity (RESET). Therefore, the weight is encoded in a pair of PCM elements that operate in SET mode in a differential configuration. Non-linearities and conductance saturation are detractors for optimal performance. However, using appropriate CMOS circuit elements these detractors can be overcome and provide a possible solution for deep learning (Ambrogio et al., 2018).

It is clear that a global asymmetry term, uniform among all devices, can be fixed easily by the supporting peripheral circuits using different voltage pulses for up and down changes for the whole array without requiring a serial access to each device. However, if there is a slight device-to-device variation that causes a local asymmetry term such a compensation is not possible without breaking the parallel nature of the array operation. Given that these arrays would be fully utilized and always busy in a pipelined design to get the most performance benefits, any kind of interruption to the parallel operations may become too costly no matter how infrequent the interruption is. Therefore, the area, power and especially the time cost of these engineering solutions need to be sized properly as it may significantly reduce the benefits of using analog arrays for DNN training.

In summary, we believe that the RPU concept is a very promising candidate to accelerate the training of a range of complex deep neural networks, and our results indicate that the huge investment of putting machine learning algorithms into such hardware is warranted. However, its success strongly depends on realizing a cross-point that can change its state in a symmetrical fashion. Once the symmetry problem is overcome, the RPU concept can provide unprecedented acceleration factors reaching 10,000x compared to the digital counterparts (Gokmen and Vlasov, 2016). For a highly optimized digital hardware one can think of fitting 10s of 1000s of multiplication and summation units on a single chip. However, even these numbers look miniscule when compared to an RPU approach, as a single RPU array consisting of 4096 × 4096 cross-points can perform 16 million multiplication and summation operations all in parallel in the analog domain by using only a fraction of the chip area. Using multiple arrays simultaneously would make the throughput of analog accelerator chip even more impressive reaching 3–4 orders of magnitude larger than the digital only solutions. Therefore, large problems of interest for business applications that currently require days of training on multiple digital hardware can take only minutes using a single RPU based analog accelerators.

## Author Contributions

TG conceived the original idea. TG, MR, and WH developed methodology, analyzed and interpreted results, drafted and revised manuscript.

## Conflict of Interest Statement

The authors declare that the research was conducted in the absence of any commercial or financial relationships that could be construed as a potential conflict of interest.

## References

[B1] Agrawal S. Plimpton S. Hughart D. Hsia A. Richter I. Cox J. (2016a). “Resistive memory device requirements for a neural network accelerator,” in *JCNN: International Joint Conference on Neural Network*, Brazil.

[B2] Agrawal S. Quach T. Parekh O. Hsia A. DeBenedictis E. James C. (2016b). energy scaling advantages of resistive memory crossbar computation and its application to sparse coding. *Front. Neurosci.* 9:484. 10.3389/fnins.2015.00484 26778946 PMC4701906

[B3] Ambrogio S. Narayanan P. Tsai H. Shelby R. M. Boybat I. di Nolfo C. (2018). Equivalent-accuracy accelerated neural netrowk training using analog memory. *Nature* 558 60–67. 10.1038/s41586-018-0180-5 29875487

[B4] Burr G. Narayanan P. Shelby R. Sidler S. Boybat I. di Nolfo C. (2015). “Large-scale neural networks implemented with non-volatile memory as the synaptic weight element: comparative performance analysis (accuracy, speed, and power),” in *IEDM (International Electron Devices Meeting)*,San Fransisco, CA.

[B5] Burr G. Shelby R. Sebastian A. Kim S. Kim S. Sidler S. (2017). Neuromorphic computing using non-volatile memory. *Adv. Phys.* 2 89–124.

[B6] Chang S. Zhang Y. Han W. Yu M. Guo X. Tan W. (2017). Dilated recurrent neural networks. arXiv:1710.02224.

[B7] Chen P. Lin B. Wang I. Hou I. Ye J. Vrudhula S. (2015). “Mitigating effects of non-ideal synaptic device characteristics for on-chip learning,” in *ICCAD ’15 IEEE/ACM International Conference On Computer-Aided Design*, Austin, TX.

[B8] Chen Y. Li J. Xiao H. Jin X. Yan S. Feng J. (2017). Dual path networks. arXiv:1707.01629.

[B9] Chilimbi T. Suzue Y. Apacible J. Kalyanaraman K. (2014). Project adam: Building an efficient and scalable deep learning training system. *OSDI* 14 571–582.

[B10] Cho K. van Merrienboer B. Gulcehre C. Bahdanau D. Bougares F. Schwenk H. (2014). “Learning phrase representations using RNN encoder-decoder for statistical machine translation,” in *Proceedings of the 2014 Conference on Empirical Methods in Natural Language Processing (EMNLP)*, Doha.

[B11] Chung J. Gulcehre C. Cho K. Bengio Y. (2015). “Gated feedback recurrent neural networks,” in *Proceedings of the 32nd International Conference on Machine Learning (ICML)*, Lille.

[B12] Coates A. Huval B. Wang T. Wu D. Ng A. (2013). “Deep learning with COTS HPC systems,” in *ICML’13 Proceedings of the 30th International Conference on International Conference on Machine Learning* Vol. 28 Atlanta, GA.

[B13] Collobert R. Weston J. Bottou L. Karlen M. Kavukcuoglu K. Kuksa P. (2012). Natural language processing (Almost) from scratch. *J. Mach. Learn. Res.* 12 2493–2537.

[B14] Dean J. Corrado G. Monga R. Chen K. Devin M. Le Q. (2012). “Large scale distributed deep networks,” in *NIPS’12 Proceedings of the 25th International Conference on Neural Information Processing Systems* Vol. 1 Lake Tahoe, NV, 1223–1231.

[B15] Emer J. Sze V. Che Y. (2016). “Tutorial on hardware architectures for deep neural networks,” in *IEEE/ACM International Symposium on Microarchitecture (MICRO-49)*, Cambridge, MA.

[B16] Fuller E. El Gabaly F. Leonard F. Agarwal S. Plimpton S. Jacobs-Gedrim R. (2017). Li-ion synaptic transistor for low power analog computing. *Adv. Mater.* 29:1604310. 10.1002/adma.201604310 27874238

[B17] Gokmen T. Onen M. Haensch W. (2017). Training deep convolutional neural networks with resistive cross-point devices. *Front. Neurosci* 11:538. 10.3389/fnins.2017.00538 29066942 PMC5641314

[B18] Gokmen T. Vlasov Y. (2016). Acceleration of deep neural network training with resistive cross-point devices. *Front. Neurosci.* 10:333. 10.3389/fnins.2016.00333 27493624 PMC4954855

[B19] Gupta S. Agrawal A. Gopalakrishnan K. Narayanan P. (2015). “Deep learning with limited numerical precision,” in *ICML’15 Proceedings of the 32nd International Conference on International Conference on Machine Learning* Vol. 37 Lille.

[B20] Gupta S. Zhang W. Wang F. (2017). “Model accuracy and runtime tradeoff in distributed deep learning: a systematic study,” in *Proceedings of the 26th International Joint Conference on Artificial Intelligence (IJCAI)*, (Melbourne, SA: AAAI Press).

[B21] He K. Zhang X. Ren S. Sun J. (2015). “Delving deep into rectifiers: surpassing human-level performance on imagenet classification,” in *2015 IEEE International Conference on Computer Vision (ICCV)*, Washington, DC.

[B22] Hinton G. Deng L. Dahl G. Mohamed A. Jaitly N. Senior A. (2012). Deep neural networks for acoustic modeling in speech recognition: The shared views of four research groups. *IEEE Signal. Proc. Mag.* 29 82–97. 10.1109/MSP.2012.2205597

[B23] Hochreiter S. Schmidhuber J. (1997). Long short-term memory. *Neural Comput.* 9 1735–1780. 10.1162/neco.1997.9.8.1735 9377276

[B24] Jouppi N. P. Young C. Patil N. Patterson D. Agrawal G. Bajwa R. (2017). “In-datacenter performance analysis of a tensor processing unit,” in *ACM/IEEE 44th Annual International Symposium on Computer Architecture (ISCA)* Vol. 2017 Toronto, ON, 1–12.

[B25] Jozefowicz R. Vinyals O. Schuster M. Shazeer N. Wu Y. (2016). Exploring the limits of language modeling. arXiv:1602.02410.

[B26] Karpathy A. Fei-Fei L. (2015). “Deep visual-semantic alignments for generating image descriptions,” in *Conference on Computer Vision and Pattern Recognition*, Long Beach, CA. 10.1109/TPAMI.2016.2598339 27514036

[B27] Karpathy A. Johnson J. Fei-Fei L. (2016). “Visualization and understanding recurrent networks,” in *ICLR*, Vancouver, BC.

[B28] Kim S. Gokmen T. Lee H. Haensch W. E. (2017). “Analog CMOS-based resistive processing unit for deep neural network training,” in *IEEE 60th International Midwest Symposium on Circuits and Systems (MWSCAS)*, Boston, MA.

[B29] Krizhevsky A. Sutskever I. Hinton G. (2012). “Imagenet classification with deep convolutional neural networks,” in *Conference on Neural Information Processing Systems (NIPS)*, Montreal, QC, 1097–1105.

[B30] LeCun Y. Bengio Y. Hinton G. (2015). Deep learning. *Nature* 521 436–444. 10.1038/nature14539 26017442

[B31] LeCun Y. Bottou L. Bengio Y. Haffner P. (1988). Gradient-based learning applied to document recognition. *Proc. IEEE* 86 2278–2324. 10.1109/5.726791

[B32] Li Y. Kim S. Sun X. Solomon P. Gokmen T. Tsai H. (2018). “Capacitor-based cross-point array for analog neural network with record symmetry and linearity,” in *Symposium on VLSI*, New York, NY.

[B33] Lipton Z. C. Berkowitz J. Elkan C. (2015). A critical review of recurrent neural networks for sequence learning. arXiv:1506.00019.

[B34] Prezioso M. Merrikh-Bayat F. Hoskins B. Adam G. Likharev A. Strukov D. (2015). Training and operation of an integrated neuromorphic network based on metal-oxide memristors. *Nature* 521 61–64. 10.1038/nature14441 25951284

[B35] Ravanelli M. Brakel P. Omologo M. Bengio Y. (2017). “A network of deep neural networks for distant speech recognition,” in *Acoustics, Speech and Signal Processing (ICASSP)*, Calgary, AB.

[B36] Rumelhart D. Hinton G. Williams R. (1986). Learning representations by back-propagating errors. *Nature* 323 533–536. 10.1038/323533a0

[B37] Sodani A. (2015). “Knights landing (KNL): 2nd generation intel xeon phiprocessor,” in *Hot Chips 27*, Cupertino, CA.

[B38] Srivastava N. Hinton G. Krizhevsky A. Sutskever I. Salakhutdinov R. (2014). Dropout: a simple way to prevent neural networks from overfitting. *J. Mach. Learn. Res.* 15 1929–1958.

[B39] Wu Y. (2016). Google’s neural machine translation system: bridging the gap between human and machine translation. arXiv:1609.08144.

[B40] Zaremba W. Sutskever I. Vinyals O. (2014). Recurrent neural network regularization. arXiv:1409.2329.

